# Effectiveness and Cost-Effectiveness of Occupation-Based Occupational Therapy Using the Aid for Decision Making in Occupation Choice (ADOC) for Older Residents: Pilot Cluster Randomized Controlled Trial

**DOI:** 10.1371/journal.pone.0150374

**Published:** 2016-03-01

**Authors:** Hirofumi Nagayama, Kounosuke Tomori, Kanta Ohno, Kayoko Takahashi, Kakuya Ogahara, Tatsunori Sawada, Sei Uezu, Ryutaro Nagatani, Keita Yamauchi

**Affiliations:** 1 Department of Occupational Therapy, Kanagawa University of Human Services, Kanagawa, Japan; 2 Graduate Course of Health and Social, Kanagawa University of Human Services, Kanagawa, Japan; 3 School of Allied Health, Department of Occupational Therapy, Kitasato University, Kanagawa, Japan; 4 IMS Itabashi Rehabilitation Hospital, Tokyo, Japan; 5 Naha City Ajya Complex Welfare Facility, Okinawa, Japan; 6 Graduate School of Health Management, Keio University, Kanagawa, Japan; Vanderbilt University, UNITED STATES

## Abstract

**Background:**

Care-home residents are mostly inactive, have little interaction with staff, and are dependent on staff to engage in daily occupations. We recently developed an iPad application called the Aid for Decision-making in Occupation Choice (ADOC) to promote shared decision-making in activities and occupation-based goal setting by choosing from illustrations describing daily activities. This study aimed to evaluate if interventions based on occupation-based goal setting using the ADOC could focus on meaningful activities to improve quality of life and independent activities of daily living, with greater cost-effectiveness than an impairment-based approach as well as to evaluate the feasibility of conducting a large cluster, randomized controlled trial.

**Method:**

In this single (assessor)-blind pilot cluster randomized controlled trial, the intervention group (ADOC group) received occupational therapy based on occupation-based goal setting using the ADOC, and the interventions were focused on meaningful occupations. The control group underwent an impairment-based approach focused on restoring capacities, without goal setting tools. In both groups, the 20-minute individualized intervention sessions were conducted twice a week for 4 months.

**Main Outcome Measures:**

Short Form-36 (SF-36) score, SF-6D utility score, quality adjusted life years (QALY), Barthel Index, and total care cost.

**Results:**

We randomized and analyzed 12 facilities (44 participants, 18.5% drop-out rate), with 6 facilities each allocated to the ADOC (n = 23) and control (n = 21) groups. After the 4-month intervention, the ADOC group had a significantly greater change in the BI score, with improved scores (P = 0.027, 95% CI 0.41 to 6.87, intracluster correlation coefficient = 0.14). No other outcome was significantly different. The incremental cost-effectiveness ratio, calculated using the change in BI score, was $63.1.

**Conclusion:**

The results suggest that occupational therapy using the ADOC for older residents might be effective and cost-effective. We also found that conducting an RCT in the occupational therapy setting is feasible.

**Trial Registration:**

UMIN Clinical Trials Registry UMIN000012994

## Introduction

Older people with a purpose in life have lower mortality [1], higher sense of well-being [2], and better maintenance of functional status, including a reduced risk of developing impairments in basic and instrumental activities of daily living (ADL) and mobility disability [3,4]. Having a purpose in life influences a person’s engagement in health-promoting behaviors, thereby indirectly affecting health [3], and the belief that one’s life is meaningful and goal-directed is associated with greater participation in physical activity [4].

An observational study conducted in a care home for older people that recorded activity in 10-minute intervals over a 16-hour period found that the residents spent most of their time sitting (97% of observations) and not engaged in activity (60.7% of observations); social interaction between residents or with a caregiver occurred infrequently (10.7% of observations) [5]. Therefore, care-home residents spend the majority of their time inactive, with low levels of interaction with staff [5,6] and are dependent on staff to engage in daily occupations [6].

Occupational therapy is an occupation-based and client-centered health profession aimed at promoting health and well-being through a variety of everyday activities important to the client [7]. Occupational therapy evaluation and interventions should be based on an occupation (occupation-based approach) that includes purposeful and meaningful activities for an individual and the social context, rather than focusing on impairments or body structure (impairment-based approach) [8,9]. Recently, we developed an iPad (Apple Inc, Cupertino, CA, USA) application called the Aid for Decision-making in Occupation Choice (ADOC) to promote shared decision-making in activities and participation level (occupation)-based goal setting by choosing from illustrations describing daily activities [10,11]. Our case studies showed that older inpatients and the occupational therapist could identify meaningful activities using the ADOC, even if they had aphasia[12] or dementia [13](Mini-Mental State Examination [MMSE] >8 points [14]).

Evidence for the benefit of rehabilitation services in care home residents is inconclusive [15],　 and the effects and cost-effectiveness of an occupation-based approach to occupational therapy using the ADOC compared with an impairment-based approach are unknown. We hypothesized that interventions based on occupation-based goal setting using the ADOC could focus on meaningful activities (occupation-based approach) that could improve quality of life (QOL) and independent ADLs, resulting in greater cost-effectiveness than an impairment-based approach. This study aimed to compare the effectiveness, including cost-effectiveness, of the occupation-based approach versus the impairment-based approach for older residents and to determine the feasibility of conducting a large cluster randomized controlled trial in this setting.

## Methods

### Study design

This study was a single-blind (assessor blind) cluster randomized controlled trial. It is difficult to blind the occupational therapist, and bias could occur if the same therapist intervened with both groups. Therefore, we chose the cluster randomized trial design to reduce between-group treatment bias. The protocol of this study was approved by the ethical committee of the School of Allied Health Science, Kitasato University (2012–032) (complete data: April 28, 2013) (S1 File). This study was registered in the UMIN Clinical Trials Registry (UMIN000012994) (Data of registration: January 28, 2014) (S2 File and S3 File).

This study started (participant recruitment) after approval of the Ethical Review Board. Because the names of the participating facilities were needed for the institutional review board protocol application, facilities were recruited prior to Ethical Review Board approval. The reason of delay trial registration is that this study was pilot study, which is going to start in future. The authors confirm that all ongoing and related trials for this intervention are registered.

### Setting

The present study was conducted in geriatric health service facilities (Roken in Japan) established by the Japanese government under the long-term care insurance scheme to provide medical treatment, nursing care, and rehabilitation to older people who need assistance with ADL [16]. The national average number of occupational therapists working in a typical geriatric health service facility is 1.2 [17], and occupational therapy or physical therapy is conducted for 20 minutes twice a week with each individual.

### Facility and participant recruitment

To reduce selection bias, the facilities and participants were recruited and provided written, informed consent prior to randomization [18,19].

The geriatric health service facilities were recruited via a website and were included if they employed 1–5 occupational therapists. We recruited more than 4 facilities for each group to meet the minimum sample size requirements [20].

To recruit residents, a placard requesting volunteers was posted on the facility bulletin board, and the occupational therapists also approached the residents about participation. The inclusion criteria were stable constitutional symptoms, no major communication or cognitive deficits, and individual training twice a week. In addition, all participants had been admitted to the facility for more than three months prior to the start of the study. Participants had not used a goal-setting tool, and an impairment-based approach had been performed twice weekly. We recruited 5 participants per facility, and all participants were assessed for eligibility and baseline data. Participants were excluded if they had an MMSE score <10, a communication deficit, cardiac or progressive disease, or were judged unable to receive occupational therapy by a physician. There were no limitations regarding disease type because residents in geriatric health facilities typically have various diseases and disability conditions.

The duration of recruitment of facilities was from September 25, 2012 to June 30, 2013. Participants were recruited from July 1, 2013 to August 30, 2013. We standardized time of the intervention initiation and termination of all facilities. (The start date of the intervention is October 1, 2013, the end date was the January 31, 2014)

### Randomization and blinding

Cluster randomization was conducted by a research assistant that was independent from the research team using a random number generator in Microsoft Excel. Geriatric health service facilities were randomly assigned (1:1) to either the ADOC group or control group.

The assessors were not aware of the study protocol and were blinded to group allocation. To blind the study participants as much as possible, the therapists were not allowed to inform the participants of their group allocation. The statistician was also blinded to the group allocation for data analysis.

### Goal setting and intervention

In the experimental (ADOC) group, we educated a representative of the occupational therapists in each facility regarding the use of the ADOC and occupation-based approach (8 hours each for 2 days for a total of 16 hours). The participants and occupational therapists each used the ADOC to identify meaningful occupations from 95 illustrations of daily occupations based on the activities and participations listed in the International Classification of Functioning, Disability and Health (ICF) [21]. Then, the participants and occupational therapists set goals and prioritized the occupations. The occupational therapists observed each participant performing the selected occupations and assessed their occupational performance. The interventions were task-specific and used meaningful occupations (e.g., using chopsticks to eat, cooking, knitting) during training to acquire occupational skills.

In the control group, evaluations were focused on patient impairment (i.e., bodily functions and structures from the ICF) that was identified through physical and cognitive testing. The therapists did not use goal-setting tools such as the Canadian Occupational Performance Measure [22] or Goal Attainment Scaling [23]. The interventions focused on restoring capacities (e.g., muscle strength exercises and cognitive training). These interventions are the most common type of occupational therapy for older residents in geriatric health service facilities in Japan.

The interventions were conducted with each individual twice a week for 4 months (20-minute sessions each for a total of 32 sessions). The length of the intervention was based on a prior case study (data not shown).

### Main outcomes

The Short Form-36 (SF-36) was used to measure QOL [24,25]. This 36-item self-report measure was designed to measure perceived health status in both the general and specific health populations. We converted the SF-36 results to the six multi-level dimensions of the SF-6D and calculated a utility score [26,27], which was used to calculate quality adjusted life years (QALY). The Barthel Index (BI) was used to measure ADLs; the BI is a 10-item scale that is scored from 0 to 100 in 5-point increments [28]. Although we included Occupational Performance Autonomy scale as outcome measure in our protocol, the result is not shown in this manuscript. The reason is that this scale was made for this study and its reliability and validity in still in progress.

We collected and calculated all direct care and medical costs, classified as either long-term care insurance costs or non-insurance costs, at the participant level using receipts collected by the facility’s study collaborator during the 4-month study period. Long-term care insurance costs included unit costs, consultation by a physician, and occupational therapy costs. Non-insurance costs included the daily cost of living, specialty room costs, additional medical costs (consultations at other hospitals), and occupational therapy material costs (e.g., assistive devices). Total costs were converted US dollars using average currency conversion rates (http://www.x-rates.com/) for the month of data collection (Feb 2014, 0.008419 US Dollar per 1 Japanese Yen).

### Sample size

Because the results of this pilot study were intended to calculate intracluster correlation coefficients (ICC) and estimate the ideal sample size for future trials, formal sample size calculations were not appropriate for this study [29]. We were confident that 12 facilities, with 5 residents each, would participate; as a result, 6 clusters and 30 participants per group were used, with a total of 60 participants.

### Statistical analysis

To confirm the validity of the randomization, we compared the baseline characteristics using two-tailed independent *t*-tests or Mann-Whitney U tests for continuous, ordinal data and Chi-square tests for categorical data. The following primary outcomes were assessed 4 months later and compared between the ADOC and control groups: change in SF-36 score, change in SF-6D utility score, QALY, change in BI score, and total cost during the study period. Analyses were performed with Stata (version 13) software using mixed multilevel procedures. Primary outcomes were analyzed using mixed effects multilevel regression analysis to compare outcome measures between the ADOC and control groups at 4 months. The analysis was adjusted by facility (random effect covariate) and baseline outcome measures (Barthel index, SF-36, and SF-6D score). QALY and total cost were evaluated using mixed effects multilevel regression, with adjustments for geriatric health service facilities (random effect covariate).ICCs were calculated to confirm the correlation in each cluster. Furthermore, the effect sizes (Cohen’s *d*) and 95% confidence intervals (CIs) were calculated. All outcomes were assessed according to intention-to-treat analysis.For all analyses, P < 0.05 was considered statistically significant.

Cost-effectiveness analysis was conducted using total costs and effectiveness (i.e., cost per unit increase in BI score), and the cost-effectiveness ratio was calculated using the QALY and change in BI score. If there was a significant difference between the groups, the incremental cost was calculated per outcome measure (incremental cost-effectiveness ratio [ICER]), relative to the control group. The ICER is the incremental increase in cost per incremental increase in outcome effect in comparison with the control group. The ICER equation is ICER = [Cost_t_ − Cost_c_]/[Outcome_t −_ Outcome_c_], where Cost_t_ and Cost_c_ are the total costs for the ADOC group and the control group, respectively, and Outcome_t_ and Outcome_c_ are the significant difference between the groups in outcome measure for the ADOC group and control groups, respectively [30].

## Results

### Trial recruitment and completion rates

**Fig 1:**
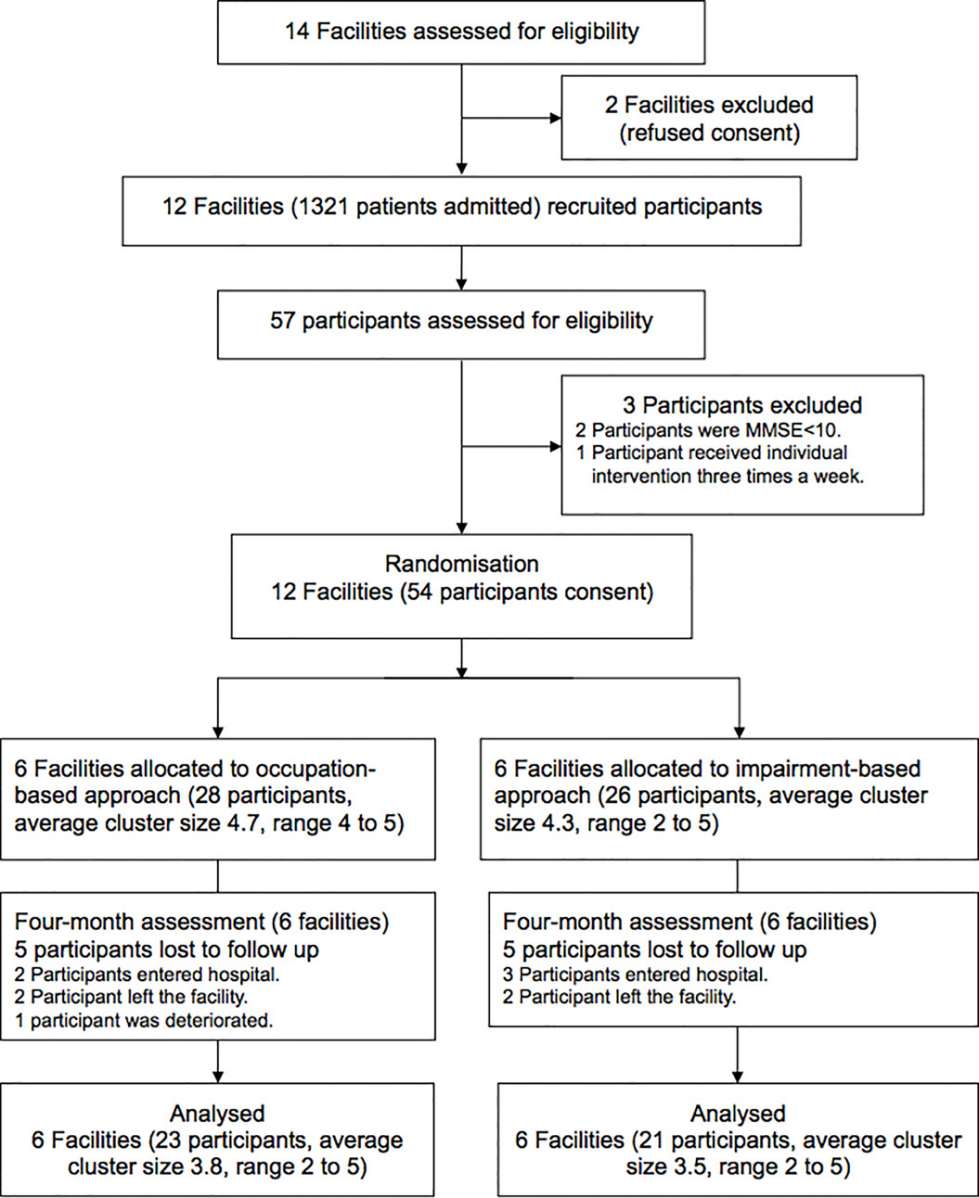
Flow diagram of recruitment of geriatric health service facilities and older residents. MMSE; Mini-Mental State Examination, ADOC; Aid for Decision-making in Occupation Choice.

The flow diagram for the CONSORT extension for cluster-randomized trials is shown in Fig 1 [31]. Of the 14 facilities that responded, 2 declined consent. The remaining 12 facilities (54 participants) were randomized to the 2 groups, with 6 facilities allocated to the ADOC group (28 participants, average cluster size 4.7, range 4–5) and 6 facilities allocated to the control group (26 participants, average cluster size 4.3, range 2–5).

Five participants each in the ADOC (2 admitted to the hospital, 2 discharged, 1 deteriorated) and control (3 admitted to the hospital, 2 discharged) groups dropped out (total 10 participants, 18.5%). The final analysis included 12 facilities and 44 participants (ADOC, 6 facilities, 23 participants, average cluster size 4.7, range 4–5; control, 6 facilities, 21 participants, average cluster size 4.3, range 2–5).

### Baseline characteristics and goal setting

There were no significant differences in the baseline participant characteristics between the groups, except for the proportion of each gender (Table 1). The number of participants who set occupation-based goals was 22/28 (78.6%) in the ADOC group and 5/26 (19.2%) in the control group (Table 2). In the ADOC group, the highest percentage of participants set a hobby as a goal, and in the control group, the highest percentage of participants set basic activities such as mobility as a goal. Thus, ADOC was able to promote occupation-based goal setting.

**Table 1:** Baseline characteristics of the older residents of geriatric health service facilities.

Characteristics	Total (n = 54)	ADOC group (6 facilities, n = 28)	Control group (6 facilities, n = 26)	P-value
Age (years), mean (SD)	82.10 (9.31)	82.50 (9.66)	85.80 (8.78)	0.20**
Gender, n (%)				
Men	8 (14.8)	7 (25.0)	1 (3.8)	0.03*
Women	46 (85.2)	21 (75.0)	25 (96.2)	
MMSE, mean (SD)	22.00 (4.18)	22.57 (3.89)	21.38 (4.47)	0.30**
Main diagnosis, n (%)				
Cerebral vascular disorder	23 (43)	12 (43)	11(42)	0.66*
Hip fracture	8 (15)	4 (14)	4 (15)	
Alzheimer disease	2 (4)	1 (4)	1 (4)	
Vascular dementia	1 (2)	1 (4)	0 (0)	
Rheumatoid arthritis	2 (4)	2 (7)	0 (0)	
Vertebral compression fractures	3 (6)	1 (4)	2 (8)	
Chronic heart failure	3 (6)	2 (7)	1 (4)	
Osteoarthritis	1 (2)	1 (4)	0 (0)	
Spinal canal stenosis	2 (4)	0 (0)	2 (8)	
Other(s)	9 (17)	4 (14)	5 (19)	

ADOC, Aid for Decision-making in Occupation Choice; MMSE, Mini-Mental State Examination.

*Compared using χ^2^ tests

**Compared using Student’s *t*-tests.

**Table 2:** Number and type of goals set in both groups.

Goal	ADOC group (%) N = 28	Control group n (%) N = 26
Hobby	10 (35.7%)	3 (11.5%)
Mobility	4 (14.3%)	18 (69.2%)
Self-care	3 (10.7%)	2 (7.7%)
Social activities	3 (10.7%)	0 (0%)
Domestic life	3 (10.7%)	0 (0%)
Body function	2 (7.1%)	3 (11.5%)
Interpersonal	2 (7.1%)	0 (0%)
Communication	1 (3.6%)	0 (0%)

ADOC, Aid for Decision-making in Occupation Choice.

### Outcomes

**Fig 2:**
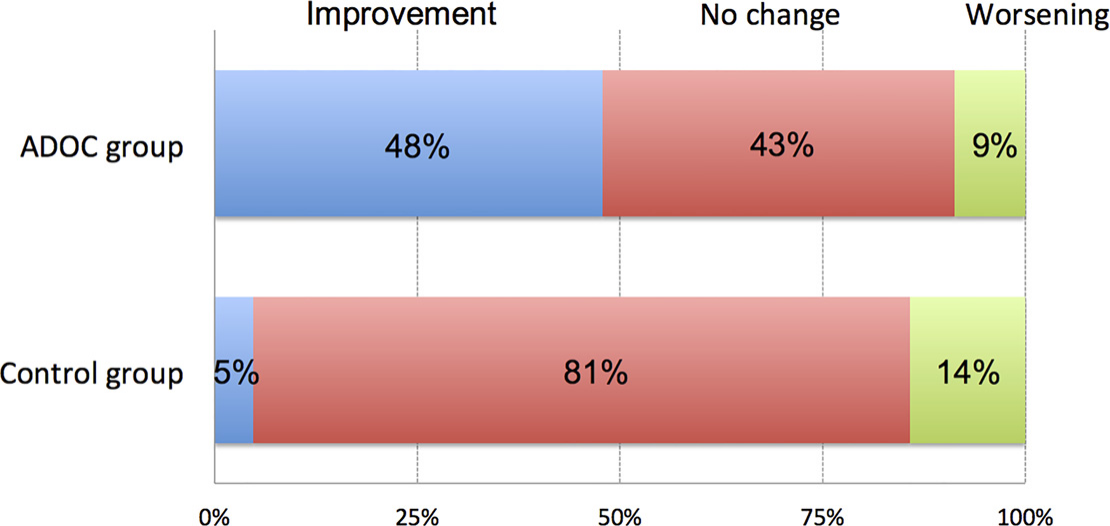
Percentage of participants stratified by changes in the Barthel Index. ADOC, Aid for Decision-making in Occupation Choice.

There were no significant differences between baseline and post-intervention outcomes in either group (Table 3). The ADOC group had a significantly higher change in the BI score than the control group (P = 0.027, 95% CI 0.72–7.19, ICC = 0.14, *d* = 0.71). Statistical power (1 − β) for the BI score was 0.63 (calculated using this result with a type I error rate of 5%, a two-sided effect).In the ADOC group, 11/23 (47.8%) participants had improved BI scores, 10/23 (43.5%) participants had stable scores, and 2/23 (8.7%) participants had decreased scores. In the control group, 1/21 (4.8%) participant had an improved BI score, 17/21 (81.0%) participants had stable scores, and 3/21 (14.3%) participants had decreased scores (Fig 2). No other significant differences between the groups were found in any of the SF-36 scores, SF-6D utility score, QALY, or total costs.

**Table 3:** Main outcomes following occupational therapy interventions lasting 4 months in older adults of geriatric health service facilities.

	ADOC group (n = 23)			Control group (n = 21)			ADOC group vs Control group				
	Mean (SD)			Mean (SD)							
	Baseline	4 months	Change score	Baseline	4 months	Change score	Adjusted coefficient	P-value	95% CI	Cohen’s *d*	ICC
Short form 36 v2											
Subscales											
Physical function	33.26 (32.43)	34.78 (32.73)	1.52 (13.85)	34.76 (27.36)	41.43 (25.94)	6.67 (18.12)	-5.42	0.245	-14.55 to 3.72	0.32	-0.03
Role physical	64.40 (39.91)	65.76 (37.59)	1.36 (36.20)	71.43 (26.11)	75.89 (35.81)	4.46 (37.09)	-5.35	0.655	-28.86 to18.14	0.08	0.15
Bodily pain	62.65 (30.49)	67.43 (34.28)	4.78 (20.44)	68.52 (29.16)	74.14 (25.48)	5.62 (18.92)	-2.01	0.718	-12.93 to 8.91	0.04	-0.13
General health	66.17 (30.50)	64.91 (24.54)	-1.26 (16.87)	70.24 (23.20)	72.33 (23.16)	2.10 (16.39)	-4.58	0.284	-12.96 to 3.79	0.20	-0.02
Vitality	66.58 (27.86)	63.86 (27.63)	-2.72 (17.36)	69.05 (24.32)	72.32 (25.96)	3.27 (21.80)	-6.62	0.223	-17.27 to 4.03	0.31	-0.13
Social functioning	72.28 (35.94)	63.59 (37.86)	-8.70 (36.82)	82.14 (23.90)	83.33 (26.61)	1.19 (36.84)	-15.27	0.163	-36.74 to 6.18	0.27	0.03
Role emotional	74.64 (36.80)	81.16 (34.10)	6.52 (36.32)	78.17 (31.46)	80.55 (32.42)	2.38 (27.02)	2.81	0.31	-14.82 to 20.45	0.13	0.11
Mental health	70.22 (22.84)	66.74 (25.48)	-3.48 (21.18)	75.95 (23.27)	75.95 (23.54)	0.00 (24.06)	-5.85	0.338	-17.79 to 6.10	0.15	-0.01
Component scale											
Physical composite	21.72 (20.94)	24.82 (20.06)	3.10 (10.96)	22.12 (17.31)	26.76 (14.72)	4.65 (14.26)	-1.82	0.614	-8.94 to 5.28	0.12	-0.06
Mental composite	62.82 (13.11)	60.19 (12.85)	-2.63 (10.33)	64.73 (13.37)	64.48 (14.47)	-0.25 (19.53)	-3.04	0.331	-9.18 to 3.09	0.21	-0.16
Role composite	42.35 (22.96)	42.08 (17.53)	-0.27 (20.32)	45.97 (13.04)	46.10 (18.86)	0.13 (19.53)	-2.65	0.600	-12.59 to 7.28	0.02	-0.02
Barthel Index	60.00 (22.96)	63.48 (22.48)	3.48 (6.11)	67.38 (23.75)	66.90 (23.64)	-0.48 (4.98)	3.65	0.027	0.41 to 6.87	0.71	0.14
SF-6D utility score	0.576 (0.119)	0.589 (0.158)	0.013 (0.105)	0.625 (0.104)	0.633 (0.124)	0.008 (0.118)	-0.004	0.898	-0.06to 0.06	0.04	-0.07
Quality adjusted life year (QALY)	0.199 (0.045)			0.221 (0.037)			-0.02	0.118	-0.05 to 0.01	0.53	0.017
Total cost (US dollars)	$11,642.7 (985.68)			$11,393.3 (1,525.6)			427.8	0.429	-633.39 to 1,488.9	0.20	0.40

ADOC, Aid for Decision-making in Occupation Choice; SD, standard deviation; CI, confidence interval; ICC, intracluster correlation coefficient.

### Cost-effectiveness

The average total cost per participant during the study period was $11,642.7 (SD 985.68) in the ADOC group and $11,393.3 (SD 1,525.6) in the control group. The cost-effectiveness ratios were $61,743.2 /QALY and $3,347.2/BI change in the ADOC group and $52,902.6/QALY and $23,925.3/BI change in the control group. The change in BI score was the only variable with a significant difference between the groups; therefore, the ICER, which was calculated using the change in BI score, was $63.1.

### Sample size calculation for future studies

Sample size was calculated using these results, with 80% power, a two-sided effect, and a type I error rate of 5%. In the present study, of the 54 participants that were eligible, 44 were included in the primary analysis. Therefore, assuming a loss to follow-up of 18.5% at 4 months, the study was powered to detect a 3.96-point difference in the BI score and 0.14-points in the ICC, with a minimum sample size of 69 participants per group (total 138 participants). For future studies, 14 facilities, with 5 participants each, are required for each group for a total of 28 facilities.

## Discussion

Key findings of this study include the following four points.

First, the ADOC was shown to be a useful and acceptable tool for both clients and occupational therapists in shared decision-making for occupation-based goal setting. The number of participants in our study who set occupation-based goals setting was 22/28 (78.6%) in the ADOC group and 5/26 (19.2%) in the control group. Goal setting with the ADOC enabled the client to easily understand the meaningful occupation, because it gave a visual cue (illustration) of ADL and other activities [10]. Levack et al. reported that, in addition improving patient outcomes measured using standardized measures, goal planning is thought to be a good framework to evaluate outcomes related to enhanced patient autonomy or patient-centered therapy [32].

Second, an occupation-based approach using the ADOC can maintain or improve ADL, compared with an impairment-based approach. The ADOC-guided training concerning meaningful occupations might enhance autonomy and purpose in life as well as maintain and improve ADL in older people as compared with standard goal setting (impairment-based approach). Similarly, a previous study reported that a greater feeling of a purpose in life is associated with maintenance of functional status, including a reduced risk of developing impairments in basic and instrumental ADL and mobility disability [3]. On the other hand, QOL did not significantly differ between the groups in spite of significant improvements in ADL. In this study, QOL was measured using SF-36 and might not reflect an effect of the occupational therapy. The primary goal of occupational therapy is to enable people to participate in the activities of everyday life [7]. Outcomes are client-driven and diverse and are measured in terms of participation, satisfaction derived from occupational participation, and/or improvement in occupational performance [7,33,34]. Therefore, interventions based on occupation-based goal setting using the ADOC may prove to be more effective if evaluation includes outcome measures using a specific occupation satisfaction scale such as the Canadian Occupational Performance Measure [22].

Third, interventions based on occupation-based goal setting using the ADOC had greater potential for cost-effectiveness than an impairment-based approach. Because of a longer follow-up period and less assistance for ADL, the total costs may decrease, although the total cost did not differ between groups for the 4-month period. Furthermore, the decision to implement the occupation-based approach using the ADOC might depend on the value placed on the impairment-based approach by health care insurance, compared with the ICER of $63.1 per change in BI score in the present study. However, the cost of the occupation-based approach might readily be accepted because it would likely be considered relatively inexpensive and, therefore, cost-effective compared with the impairment-based approach.

Finally, this pilot study provides important information regarding the study design for future studies, thus this study protocol has high feasibility. The estimated sample size, calculated using the BI results (with a loss to follow-up ratio of 18.5% at 4 months, was powered to detect a 3.96-point difference in the BI score and 0.14-point difference in the ICC), was 138 participants (69 in each group), which is much smaller than that in a previous study (330 participants) [35]. Furthermore, there are few studies regarding the cost-effectiveness of occupational therapy in older people [36].We believe that this pilot study contributes to future occupational therapy studies and that the findings can be easily applied to daily clinical practice.

### Study limitations

There are several limitations in this study. First, the method of facility recruitment through the website may have resulted in selection bias. All of the facilities were likely already interested in occupational therapy using the ADOC, and the facilities in the control group may have performed occupation-based goal setting during the study. Second, the validity of the intervention in the ADOC group may have been affected if occupation-based goal setting was not completely implemented. Moreover, we educated only some of the occupational therapists as the facilities were located throughout the country, making it difficult to train all therapists involved. In future studies, the training that the therapists receive for interviewing and goal setting using the ADOC may need to be improved. Third, there was a lack of opportunity costs and follow-up analysis. With a longer follow-up period, less assistance may be required with ADL following this type of intervention, and all costs, including opportunity costs may be decreased further in the ADOC group. These considerations will be incorporated in our future, larger studies that we are currently planning.

## Conclusion

This pilot study demonstrated the feasibility of an RCT in occupational therapy, and the findings can be easily applied to daily clinical practice. Moreover, the effectiveness and cost-effectiveness of occupational therapy in older residents using the ADOC were shown.

## Supporting Information

S1 File Study Protocol (Japanese). (PDF)

S2 File CONSORT 2010 Checklist. (DOCX)

S3 File Study Protocol (English). (PDF)
