# Supplementary material for: Effectiveness and Cost-Effectiveness of Occupation-Based Occupational Therapy Using the Aid for Decision Making in Occupation Choice (ADOC) for Older Residents: Pilot Cluster Randomized Controlled Trial
Source: PLoS One. 2016 Mar 1;11(3):e0150374. doi: 10.1371/journal.pone.0150374 (PMC4773241; doi:10.1371/journal.pone.0150374)
Supplement: S1 File — (PDF) [file pone.0150374.s001.pdf]

様式第二号

学部長
北里

平成２５年４月８日
April 8, 2013

受 付 番 号：2012－032
利益相反申請受付番号：
申請者
所属・職名：OT・助教
氏　　　名：長山洋史　殿

Hirofumi Nagayama
Assistant Proffessor
Department of Occupational Therapy

北里大学医療衛生学部
研究倫理審査委員会
委員長　田ヶ谷浩邦　印

審 査 結 果 通 知 書

Title: Effect and cost-effectiveness analysis of occupational based occupational therapy for elderly people in geriatric facility:randmized controlled trial

申請課題名：介護老人保健施設における作業に焦点を当てた作業療法の効果と費用効果分析～無作為化比較試験による検証～

先に申請のあった上記課題につき、研究倫理審査委員会での審議の結果を以下のとおり通知します。

（１）非該当
（２）承認　　Approval
（３）条件付承認
（４）変更の勧告
（５）不承認

意見：

指針：上記申請課題を研究実施計画書に則って行うことを承認します。

追記：医療衛生学部長が交代した際には、文書中、当該氏名を変更してください。なお、研究終了後は研究終了報告書（様式三号）を研究倫理審査委員会あてに提出すること。

以上

様式一号

※重要※　申請書の作成を始める前に、まず「倫理申請の手引き」を読むこと。
　　　　　また疑問点は「Q＆A集」で解決できる場合があります。

==

倫理審査申請書

受付番号	ー
受付年月日	平成　　年　　月　　日

医療衛生学部
学部長 北里 英郎 殿

下記の研究について倫理審査を申請いたします。なお申請にあたりましては、文部科学省・厚生労働省から通達された「疫学研究に関する倫理指針」および「臨床研究に関する倫理指針」の趣旨に沿って研究を実施することを申し添えます。

予備審査委員氏名	
予備審査年月日	平成　　年　　月　　日
修正確認日	平成　　年　　月　　日

確認印

【1】研究課題名	介護老人保健施設における作業に焦点を当てた作業療法の効果と費用効果分析～無作為化比較試験による検証～
【2】　申請者<br>(研究責任者)	所属部局・職　作業療法学専攻　助教　　氏名　　長山　洋史　　印<br>講習会受講歴（有）・無　　　　　　　　(職員番号：　80163　　)
	電話：042－778-9694　　　E-mail：nagayama@kitasato-u.ac.jp
【3】　所属長<br>(申請者と異なる場合)	所属部局・職　作業療法学専攻　教授　　　氏名　福田　倫也　印
	電話：042－778－9701　　　E-mail：mifukuda@kitasato-u.ac.jp

【4】　学内共同研究者　氏名・職員番号等<br>講習会受講履歴（有）・無	所属　部局　・　職　(又は学年)
成田香代子　(職員番号14558)	医療衛生学部作業療法学専攻　講師

【5】　学外共同研究者　氏名・講習会受講歴<br>講習会受講履歴　有・無	所属　機関　・　部局　・　職(又は学年)
友利幸之介　　講習会受講履歴（有）・無<br>小河原格也　　講習会受講履歴（有）・無<br>大野勘太　　講習会受講履歴　有・（無）<br>山内慶太　　講習会受講履歴　有・（無）<br><br>なお、無しの者については、ICR臨床研究入門（ICRweb、http://www.icrweb.jp/）にて受講し、終了証を提出するよう求める。	神奈川県立保健福祉大学　保健福祉学部　講師<br>神奈川県立保健福祉大学　保健福祉学部　講師<br>麻生総合病院　作業療法士<br>慶応義塾大学大学院健康マネジメント研究科　教授

【6】　研究実施期間	承認日～　26年3月1日
【7】　申請する研究の分類その１	［○］臨床研究［］疫学研究　（［］に○を入れる）
【8】　申請する研究の分類その２	「倫理申請の手引き」の表で該当するアルファベット／番号=B

【9】　研究目的

1）介護老人保健施設の現状
　介護老人保健施設では入所者100名に対して、理学療法士もしくは作業療法士1名配置が義務付けられているものの、全体的に療法士のマンパワー不足は否めない状況である。そのため現状は、対象者の個々のレベルで実践する作業療法の専門性的な関わりが困難な状況となっている。
2）トップダウンアプローチ
　近年、作業療法において、トップダウンアプローチである作業に焦点を当てた実践（Occupation-based practice: OBP）が着目されている。通常、OBPは患者や対象者（クライエント：CL）中心の介入プロセス（演繹的推論）によって展開される。つまり、CLの生活のニーズを理解した上で、CLにとって重要な作業を特定する。そしてその作業場面を観察し、作業が困難な原因について評価と介入を行う。この様に、演繹的に生活状況から作業困難に対し介入することをトップダウンアプローチと呼ばれる。
3）ボトムアップアプローチ
　一方、対象者の障害や機能面に着目し、それらに対し評価、介入し、生活状況を改善して行くといったアプローチである。現在の臨床場面においては、CLや作業療法士自身も作業より障害の改善に意識が向いていることなどから、ボトムアップの介入プロセスが主流となっている（帰納的推論）。
　この2つのアプローチは、状況において使い分けることが妥当であると考えられる。ただし作業療法業界における全体的な傾向としては、トップダウンアプローチこそが、作業療法の専門性を活かしたアプローチであることが示唆されている。トップダウンアプローチでは、最初にCLにとって重要な作業を特定し、その作業を中心にアプローチを展開していくため、作業の特定はアプローチ全体を方向づける非常に重要なプロセスといえる。しかし、半構成的面接法では、面接法ごとに背景にある理論や面接技術の習得が必要となり、トップダウンアプローチを実践する上での障壁となることがある。
　そこで我々は、比較的容易にCLにとって重要な作業を特定することができるiPadアプリ（Aid for Decision-making in Occupation Choice; ADOC）を開発した。作業療法を実施していたCL100名による予備調査では、ADOCは特別な理論やマニュアルが無くとも、iPadのインターフェイスの指示にしたがっていくことで、意味のある作業が選択され、そのプロセスをシェアするShared decision-making（SDM）が促進される可能性が示唆された。また作業に焦点を当てたSDMを通して、その後の作業療法介入は、スムーズにトップダウンの介入が導入できることも経験した。
　以上の様に、トップダウンアプローチの有用性は指摘されているものの、ボトムアップアプローチ（通常の訓練）との比較や、多施設での無作為化比較試験などが行われていない現状がある。
　今回、介護老人保健施設において、上記2つの介入方法の違いによる生活の質（Quality of life: QOL）改善効果の差と費用効果について、クラスター型多施設間無作為化比較試験（Cluster randomized control trial; RCT）によって検証することが目的である。

【10】　研究方法

1）デザイン
　クラスター型多施設間無作為化比較試験（Cluster randomized control trial; RCT）とする。クラスター型多施設間無作為化比較試験とは、施設を無作為に介入群（本研究ではトップダウンアプローチ群）とコントロール群（本研究ではボトムアップアプローチ群）に分けその効果について検討するデザインである。通常のRCTでは、個々人の対象者を無作為化するのに対し、クラスター型多施設間無作為化比較試験では、施設を無作為化するため同じ施設内での混入（contamination）が防止できるといった点が利点である。Consolidated Standards of Reporting Trials：CONSORTに従ったフローチャートと、研究代表者、研究分担者、研究協力者などの役割や研究手順を下に示す。また研究に関わるものの定義は以下の通りである。

研究代表者
長山洋史。研究全体の統括を行う。

研究参加者
研究に協力が得られた患者やクライエント。

研究分担者
施設所属の作業療法士。研究代表者と連絡を取り合い、所属施設のデータ管理などを行う。研究協力者Aを兼ねる。

研究協力者A
施設所属の作業療法士。研究参加者への同意を得た上で、面接、介入を行う。

研究協力者B
施設外の作業療法士で、研究参加者がどちらの群に振り分けられたか盲検化された状態でアウトカムの評価を行う。

割付け担当者
大野勘太。研究分担者と連絡をとりながら割付けを行う。

統計担当者
長山洋史。盲検化された状態で統計処理を行う。

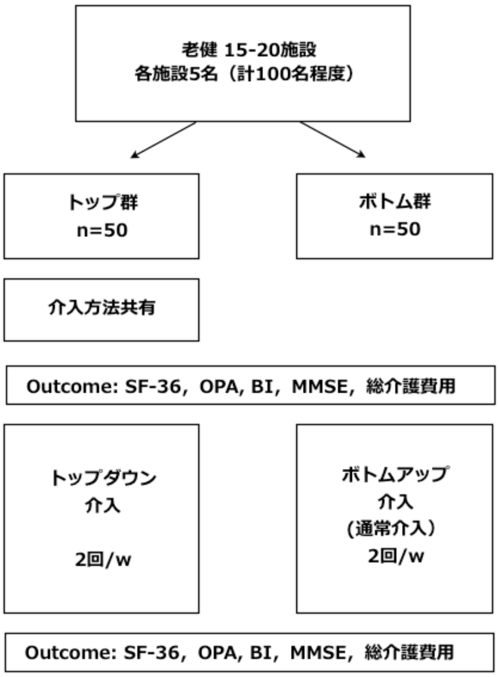

図 1. 研究のフローチャート

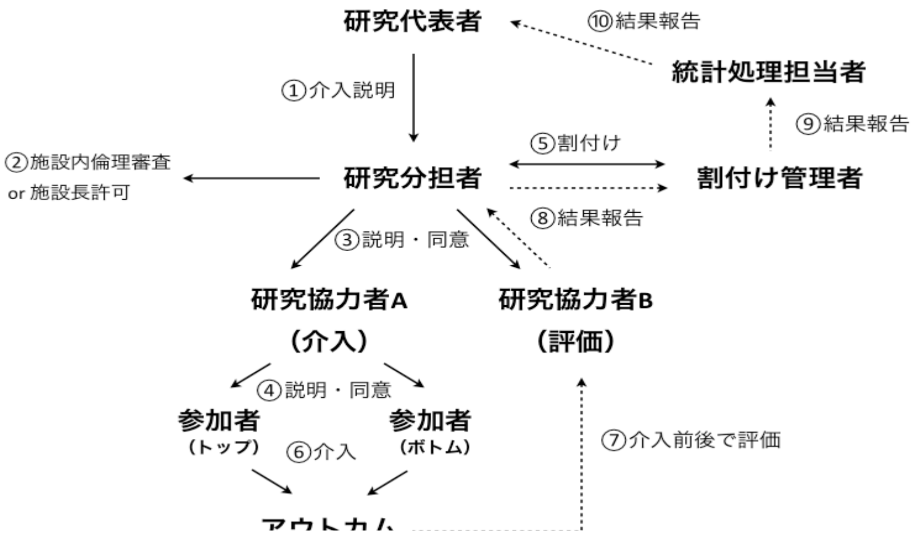

図 2. 研究に係わる者の役割

2）Setting
介護老人保健施設 14 施設以上とする。施設の包括基準として、介護老人保健施設（従来型）であり、在宅復帰支援機能加算（Ⅰ）を算定していないこと、平均要介護度 3－4、作業療法士が 1-5 人、通常ボトムアップアプローチに基づいた訓練を展開していること、とする。なお施設間のバラツキを抑える目的で、作業療法介入を行う研究協力者 A は経験 10 年未満とする。

3）対象（研究参加者）

上記介護老人保健施設入所者100名。1施設あたり5-6名を予定している。研究参加者の包括基準は、進行性の疾患はなく、全身症状が安定していること。コミュニケーション能力や認知機能に大きな問題はないこと、個別訓練を週2回実施していることとする。除外基準は、研究に参加する時点で（３ヶ月以内）MMSE20点未満の方、コミュニケーション能力に問題のある方とする。中止基準は、研究参加者やその家族が中止を希望した場合、または担当医によって研究が継続できないと判断された場合とする。

4）割付け

施設を無作為に「トップ群」と「ボトム群」に割付けする。割付けは全て研究代表者が委託した者（割付け管理者）が実施する。割り付け方法は、封筒法を用いる。2群間の例数と重症度をそろえるために、バーサルインデックスで80点以上は軽度、40点未満は重度とし、各施設で、軽度、重度をそれぞれ2-3名ずつ選出してもらう。研究参加者を極力盲検化するために、研究協力者A・Bは研究参加者がどちらの群に割り振られたか、研究参加者から聞かれない限り伝えないこととする。

5）盲検化

シングル・ブラインド化（評価者のみ盲検化。対象者や介入実施者は盲検化しない）を行う。そのためアウトカム測定は割り付けを知らない施設外の作業療法士（研究協力者B）へ委託する。統計処理および結果のレポート作成も盲検化された統計学担当者に送られる。割付けは上記のとおり割付け管理者のみ把握し、介入する作業療法士と研究代表者に対して盲検化を行う。

6）介入

トップ群の対象者は、ADOCによって特定した作業をもとに、トップダウンの作業療法を展開する。特定された作業をなるべく実際に近い形で遂行してもらい、作業療法士はそれを観察し、作業遂行の質を評価、分析する。その作業の可能化に向けて、日本作業療法士協会指定の生活行為向上プログラムを参考に、「基礎練習」、「基本練習」、「応用練習」、「社会適応練習」のいずれかを行うが、基本的には応用練習、社会適応練習を中心に（総時間数の2/3以上）取り組むこととする。このトップダウンの作業療法については、割り当て決定後、2日間の講習会およびその後のフォローアップを実施する。

一方、ボトム群は、トップ群と同じ実施時間、頻度で面接にてアセスメントした日常生活で困っていることについて、基礎練習、基本練習を中心に、応用練習へと段階を踏んだ介入を行うこととする。試験開始から4ヶ月目でアウトカム測定を行う。4ヶ月経過せずに退所した場合でもアウトカム評価を行う。

7）アウトカム

主要アウトカム

日本語版MOS 36-Item Short-Form Health Survey（特定非営利活動法人　健康医療評価研究機構にて使用登録済、使用登録完了日2012年6月29日、使用期間 ：2012年7月～2015年3月）

副次アウトカム

バーサルインデックス
OPA（Occupational performance Autonomy）
期間内に発生した総介護費並びに総医療費（レセプトデータより）
（個別加算など包括以外で発生した費用を含む）
リハビリテーションに費やした時間
転機（自宅、自宅外退所、死亡のいずれか）
入所日数

8）解析

結果の妥当性を高めるために intention to treat analysis を適用する。全てのアウトカムは、可能な限り実数で示し、95%信頼区間を求める。Quality adjusted life years（QALY）による解析では、SF-36 から効用値を換算し、QALY を算出し、期間内総介護費用並びに医療費より 1QALY あたりの費用を算出する。トップ群とボトム群の 1QALY あたりの費用を比較し、増分費用効果比（incremental cost-effectiveness ratio: ICER）を算出し、費用効果的であるかについて解析する。アウトカムと QALY の統計処理に関しては、慶応義塾大学大学院 高橋武則客員教授にアドバイスをいただく予定である。

9) その他
出版バイアスを回避するために、倫理審査終了後は UMIN 臨床試験登録システム（http://www.umin.ac.jp/ctr/index-j.htm）に登録する。

【11】 提供を受けようとする人体試料（血液、組織、体液など）および個人情報の内容

人体試料の種類と量 ：
個人情報の内容：年齢、性別、発症日、疾患名、障害名、作業療法目標、リハビリテーション実施時間、アウトカム、単位数、在所日数、退所先、総介護費用、総医療費

【12】 被験者予定数（予定人数）	被検者の集め方
患者（判断能力のある成人） ：100 名<br>患者（判断能力のない成人） ： 0 名<br>患者（20 歳未満） ： 0 名	対象者の募集については、各施設所属の研究分担者が、各施設の倫理委員会または所属長の許可を経て行う。具体的には、施設内に研究参加募集のポスターを一定期間掲示した上で、入所者自ら参加希望があれば対応する。参加希望が無ければ、包括基準を満たす入所者に対し、作業療法士より声かけを行う。その際、強制的にならないよう十分に注意する。
健常者（判断能力のある成人）： 0 名<br>健常者（判断能力のない成人）： 0 名<br>健常者（20 歳未満） ： 0 名	

【13】 研究実施場所、もしくは、人体試料および個人情報等の提供を受ける場所（複数の場合もすべて記載）

患者：研究分担者、研究協力者の所属する施設

【14】 その他の解析を行う場所と内容

学内：医療衛生学部作業療法学研究室
解析内容：群内単純集計、群間比較

学外：各研究分担者、協力施設
解析内容：データ入力のみ

【15】 研究実施、人体試料採取、個人情報収集の際の安全性、配慮等

本研究では、人権の基本である自由意思を第一優先事項とし、それを尊重することを、研究分担者が研究参加者ならびに施設関係者など、本研究の関係者へ充分説明を行う。本研究への参加は、あくまでも個人の自由意思によるものであり、いつでも参加を取り止めることが可能であること、もし途中で参加を取り止めても不利益は被らないこと、研究によって得られた個人情報は本研究のみで使用し、学会や論文などにおいて結果を公表する際に個人が同定されないよう個人情報は削除することなどを、研究分担者が説明書で充分に説明し、同意書にて研究参加を了承した方のみ実施する。各施設における対象表が破棄される前の時点で同意撤回が表明された場合は、各施設における資料とともに、ID に基づいて北里大学の匿名化データも破棄し、解析から除外する。

なお、ADOC による面接（トップ群）もしくは通常の面接（ボトム群）は、1 回 20～40 分程度である。トップ群の研究参加者は iPad 上にディスプレイされるイラストを見ながら、自分にとって重要な作業を選定し、作業療法士と一緒に目標を設定する。またボトム群の研究参加者は会話によって目標となる作業を選定し、作業療法士と一緒に目標を設定する。

・アウトカム測定について

アウトカム評価に関しては、通常作業療法で実施されている以外のアウトカム評価は、SF-36、OPA である。それ以外は作業療法での臨床場面で一般的に実施されるものである。SF-36、OPA とも聞き取りや自記式であり、それぞれ 15 分程度で終わるため、研究参加者にとっても大きな負担にはならないと推察される。ただし、研究参加者の疲労が観察された場合は中止すること、通常の訓練時間の確保を優先することなどを徹底する。

・無作為割付けに関して

本研究は無作為化比較試験であり、研究参加者は無作為にトップダウンアプローチを実施する群と、ボトムアップアプローチを実施する群に振り分けられる。そのため、研究参加者は自身の希望するアプローチを選択することはできない。ただし、振り分け後はいつでも協力を辞退すること、そして辞退しても不利益は被らないことを保証する。エンドポイントの 4 ヶ月目において、作業療法の効果において差が出てくる可能性がある。ただし、現時点においてどちらのほうが効果的か先行研究では検証されていない。今回は、海外で推奨されつつあるトップダウンアプローチを新しい介入として、その効果を検証することが目的であるが、ボトムアップアプローチは我が国では標準的に用いられている方法であり、仮にボトムアップアプローチを実施する群に振り分けられたとしても、一般的に実施されている作業療法介入以下のレベルの介入を受けるという不利益は生じない。また、ボトムアップアプローチの群に振り分けられたとしても、研究終了後に希望があればトップダウンアプローチを実施することができるようにする。

【16】　説明方法と同意（インフォームドコンセント）の取得方法　（「倫理申請の手引き」を参照して［］に○を入れる）

説明　　：［○］文書＋口頭による説明　　［］口頭のみによる説明と説明内容の記録

同意確認：［○］同意書への署名依頼　　［］口頭による確認とその記録作成

［○］情報公開による拒否機会の提供（掲示、インターネットなどの具体的方法を下に記載）

研究期間中は、施設内の掲示板等に、研究内容についての掲示を行い、施設利用者や家族がいつでも研究への研究の継続意思を確認できる機会を提供する。

［］その他（匿名化済み試料・資料の場合等を下に記載）

説明文書および同意書を添付（健常者を対象とする場合は患者用とは別の文書を用意）。掲示物等も添付。

【17】　個人情報の保護　（該当するすべての［］に○を入れる）

北里大学

匿名化法：　［］　連結可能匿名化　　［○］　連結不可能匿名化　　［］匿名化しない

個人情報を含む資料の保管法

［○］他のコンピュータと切り離されたコンピュータを使用し、外部記憶媒体（　USB　）に記憶させ、

その記憶媒体は鍵をかけて厳重に保管する。
[] 印刷または筆記による原簿、[] 撮影ビデオ、[] 写真、として鍵をかけて厳重に保管する。
[] その他（具体的に：　　　　　　　　　　　　　　　　　　　　　　）
参加施設
匿名化法：　[○]　連結可能匿名化　　　[]　連結不可能匿名化　　[] 匿名化しない
個人情報を含む資料の保管法
[○] 他のコンピュータと切り離されたコンピュータを使用し、外部記憶媒体（　USB　　）に記憶させ、その記憶媒体は鍵をかけて厳重に保管する。
[] 印刷または筆記による原簿、[] 撮影ビデオ、[] 写真、として鍵をかけて厳重に保管する。
[] その他（具体的に：

【18】　個人情報管理者

氏名:
所属・職名：
連絡先電話：

【19】　研究協力に伴う謝礼：　[] 有　　[○] 無　　([] にＯを入れる、有の場合には、その内容)

【20】　被験者に関する解析結果を当人に開示するか否か

[] 必ず開示
[] 被験者から要求があった場合のみ開示
開示の理由：
開示法：
[○] 下記の理由により開示しない　(該当するものすべてにＯを記入)
[○] 被験者に直接的利益がもたらされないから
[] この研究によって直ちに病気の原因等が解明されるわけではないから
[] 連結不可能匿名化されているから

【21】　研究終了後の人体由来試料・個人情報資料等の廃棄・保管（該当する [] すべてにＯを入れる）

人体由来試料（血液・尿など）
[] オートクレーブまたは次亜塩素酸処理の後、医療廃棄物として廃棄
[] 匿名化されたまま保管（保管場所：　　　　　　　　　　　　　　　　）
[] その他（具体的に：　　　　　　　　　　　　　　　　　　　　　　）

個人情報を含む資料
[○] コンピュータ削除ソフト（ Blancco）を使用して完全削除
[○] 外部記憶媒体（　USB　　　　）を物理的に破壊
[○] 印刷資料・写真等をシュレッダー処理後、一般ゴミとして廃棄
[] 撮影したビデオを消去
[] 匿名化されたまま保管（保管場所：　　　　　　　　　　　　　　　　）
[] その他（具体的に：　　　　　　　　　　　　　　　　　　　　　　）

【22】　他施設との共同研究　：[○] 有　　[] 無　　(該当 [] にＯを入れる、有の場合は下記項目を記載。複数の場合はまとめて別紙を作成)

施設名：
研究責任者：
担当業務：

上記施設における倫理委員会による審査状況（該当 [] にＯを入れる）
[] 承認済み　[] 審査申請中　[] 申請予定（　　年　　月頃）　[○] 倫理委員会が設置されていない。
審査申請書、被験者への説明文書、同意書、承認書等のコピーを添付。
外部研究者の所属施設に倫理委員会が設置されていない場合は、「倫理審査依頼（様式第七号）」も併せて添付。

【23】　細胞・組織バンクへの試料等の寄託：[] 有　　[○] 無　　([] にＯを入れる。有の場合はバン

クの名称)
【24】　被験者から、目的・方法などに関する研究内容の詳細（当人に関する結果は【20】に記入）について閲覧を求められた場合の対応：[〇] 閲覧可　[] 閲覧不可　([] に O を入れる。
担当者、並びに研究責任者より説明する。また、倫理審査終了後は UMIN 臨床試験登録システム (http://www.umin.ac.jp/ctr/index-j.htm) に登録し、目的、方法、デザインなどの詳細をオンライン公開する。
【25】　利益相反に関する申告– この研究に関連し、過去 2 年間に企業等の営利団体から受けた金銭的、人的、または他の助成：　[] 有　[〇] 無　([] に O を入れる。有の場合は下に記載)
1。企業等名：<br>助成金額：<br>人的、他の助成内容：<br>2。企業等名：<br>助成金額：<br>人的、他の助成内容：<br>3。企業等名：<br>助成金額：<br>人的、他の助成内容：
【26】北里から他施設または他施設から北里への試料および個人情報の移動：[〇] 有　[] 無　([] に O を入れる。有の場合は匿名化の時期と場所、運搬方法等を下記に図示。)
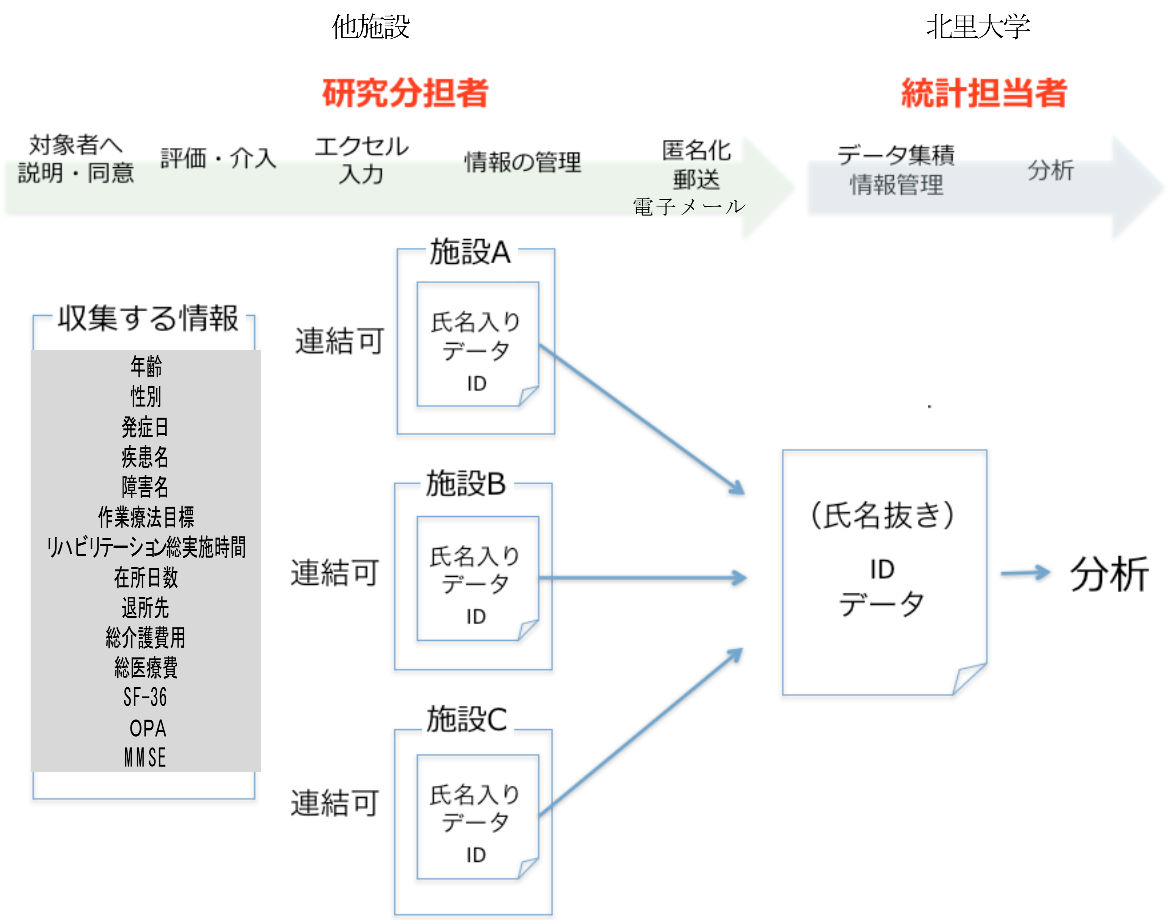
【27】　その他審査を受けるにあたり配慮すべき点、質問、要望事項等
