# Supplementary material for: Effectiveness and Cost-Effectiveness of Occupation-Based Occupational Therapy Using the Aid for Decision Making in Occupation Choice (ADOC) for Older Residents: Pilot Cluster Randomized Controlled Trial
Source: PLoS One. 2016 Mar 1;11(3):e0150374. doi: 10.1371/journal.pone.0150374 (PMC4773241; doi:10.1371/journal.pone.0150374)
Supplement: S3 File — (PDF) [file pone.0150374.s003.pdf]

UMIN CTR 臨床試験登録情報の閲覧

BACK TOP UMIN-CTRホーム 用語の説明（簡易版） 用語の説明（詳細版）--準備中 ＦＡＱ

試験進捗状況	：	試験終了/Completed
ＵＭＩＮ試験ＩＤ	：	UMIN000012994
試験名	：	介護老人保健施設における作業に焦点を当てた作業療法の効果と費用効果分析～無作為化比較試験による検証～
登録日（＝情報公開日）	：	2014/01/28
最終データ内容更新日時	：	2014/09/10 15:12:22

※ 本ページ収載の情報は、臨床試験に関する情報公開を目的として、UMINが開設しているUMIN臨床試験登録システムに提供された臨床試験情報です。

※ 特定の医薬品や治療法等については、医療関係者や一般の方に向けて広告することは目的としていません。

基本情報 (Basic information)		
項目(Item)	日本語(Japanese)	英語(English)
試験名 (Official scientific title of the study)	介護老人保健施設における作業に焦点を当てた作業療法の効果と費用効果分析～無作為化比較試験による検証～	Effect and cost-effectiveness analysis of occupational based occupational therapy for elderly people in geriatric facility:randmized controlled trial
試験簡略名 (Title of the study (Brief title))	介護老人保健施設における作業に焦点を当てた作業療法の効果と費用効果分析～無作為化比較試験による検証～	Effect and cost-effectiveness analysis of occupational based occupational therapy for elderly people in geriatric facility:randmized controlled trial
試験実施地域 (Region)	日本/Japan	

対象疾患(Condition)		
項目(Item)	日本語(Japanese)	英語(English)
対象疾患名 (Condition)	介護老人保健施設に入所している高齢所	eldely people in geriatric facility
疾患区分1 (Classification by specialty)	老年内科学/Geriatrics<br>リハビリテーション医学/Rehabilitation medicine	
疾患区分2 (Classification by malignancy)	悪性腫瘍以外/Others	

ゲノム情報の取扱い (Genomic information)	いいえ/NO

目的(Objectives)		
項目(Item)	日本語(Japanese)	英語(English)
目的1 (Narrative objectives1)	介護老人保健施設において、作業に焦点を当てた作業療法と通常のリハビリテーションの２つの介入方法の違いによる生活の質（Quality of life: QOL）改善効果の差と費用効果について、クラスター型多施設間無作為化比較試験（Cluster randomized control trial; RCT）によって検証することが目的である。	The aim of this study is investigated effect and cost-effectiveness of coccupational based occupational therapy for elderly people in geriatric facility:a cluster randmized controled trial
目的2 (Basic objectives2)	有効性/Efficacy	
目的2 -その他詳細 (Basic objectives -Others)		
試験の性質1 (Trial characteristics_1)		
試験の性質2 (Trial characteristics_2)		
試験のフェーズ (Developmental phase)		

評価 (Assessment)		
項目(Item)	日本語(Japanese)	英語(English)
主要アウトカム評価項目 (Primary outcomes)	日本語版MOS 36-Item Short-Form Health Survey	MOS 36-Item Short-Form Health Survey(japanes version)
副次アウトカム評価	バーサルインデックス<br>OPA (Occupational performance Autonomy)<br>期間内に発生した総介護費並びに総医療費	Barthel index(Activities of daliy living:ADL)

項目<br>(Key secondary outcomes)	（レセプトデータより個別加算など包括以外で発生した費用を含む）<br>リハビリテーションに費やした時間<br>転機（自宅、自宅外退所、死亡のいずれか）<br>入所日数	Occupational performance<br>Autonomy:OPA<br>Total medical cost or total care cost

基本事項 (Base)		
項目(Item)	日本語(Japanese)	英語(English)
試験の種類<br>(Study type)	介入/Interventional	

試験デザイン (Study design)		
項目(Item)	日本語(Japanese)	英語(English)
基本デザイン<br>(Basic design)	並行群間比較/Parallel	
ランダム化<br>(Randomization)	ランダム化/Randomized	
ランダム化の単位<br>(Randomization unit)	集団/Cluster	
ブラインド化<br>(Blinding)	オープンだが測定者がブラインド化されている/Open -but assessor(s) are blinded	
コントロール<br>(Control)	実薬・標準治療対照/Active	
層別化<br>(Stratification)	いいえ/NO	
動的割付<br>(Dynamic allocation)	はい/YES	
試験実施施設の考慮<br>(Institution consideration)	施設をブロックとみなしている/Institution is considered as a block.	
ブロック化<br>(Blocking)	はい/YES	
割付コードを知る方法<br>(Concealment)	中央登録/Central registration	

介入 (Intervention)		
項目(Item)	日本語(Japanese)	英語(English)
群数<br>(No. of arms)	2	
介入の目的<br>(Purpose of intervention)	治療・ケア/Treatment	
介入の種類<br>(Type of intervention)	手技/Maneuver	
介入1<br>(Interventions/Control_1)	介入群の対象者は、ADOCによって特定した作業をもとに、トップダウンの作業療法を展開する。特定された作業をなるべく実際に近い形で遂行してもらい、作業療法士はそれを観察し、作業遂行の質を評価、分析する。その作業の可能化に向けて、日本作業療法士協会指定の生活行為向上プログラムを参考に、「基礎練習」、「基本練習」、「応用練習」、「社会適応練習」のいずれかを行うが、基本的には応用練習、社会適応練習を中心に（総時間数の2/3以上）取り組むこととする。このトップダウンの作業療法については、割り当て決定後、2日間の講習会およびその後のフォローアップを実施する.	In the intervention group, clients and occupational therapists begins to share and identifiy meaningful occupations for the client by using Aid for Decision-making in Occupation Choice (ADOC). Then, occupational therapist observed the meaningful occupation and analyzed occupational performance. Interventions focused on enabling the occupation. During the care facility stay, majority interventions (had to be more than 2/3) was "Real-occupationnal practice (P-ADL)" and "Real-occupational practice (except for P-ADL)", and as appropriate, intervention in TOP group was allowed the "Basic function exercise" and "Simulated occupation practice" within the range of 1/3.
介入2<br>(Interventions/Control_2)	コントロール群の作業療法は，患者の基礎的能力（身体機能）やADLを評価する．介入は，基本的な機能訓練や，模擬的作業を用いた訓練を	In the control group, occupational therapist focus evaluations on the patient's generic abilities (i.e., boby functions and structures according to the ICF) and personal activities of daily living (P-ADL). During the care facility stay, majority interventions (had to be more than 2/3) was "Basic function exercise" and "Simulated occupation practice", and as

	2/3以上行う．実動作訓練は1/3以内とする．	appropriate, intervention in BOT group was allowed the "Real-occupationnal practice (P-ADL)" and "Real-occupational practice (except for P-ADL)" within the range of 1/3. This intervention was usual practice in all occupational therapist participated in this study (data not shown).
介入3 (Interventions/Control_3)		
介入4 (Interventions/Control_4)		
介入5 (Interventions/Control_5)		
介入6 (Interventions/Control_6)		
介入7 (Interventions/Control_7)		
介入8 (Interventions/Control_8)		
介入9 (Interventions/Control_9)		
介入10 (Interventions/Control_10)		

適格性 (Eligibility)		
項目(Item)	日本語(Japanese)	英語(English)
年齢（下限） (Age-lower limit)	60 歳/years-old 以上/<=	
年齢（上限） (Age-upper limit)	適用なし/Not applicable	
性別 (Gender)	男女両方/Male and Female	
選択基準 (Key inclusion criteria)	進行性の疾患はなく、全身症状が安定していること。コミュニケーション能力や認知機能に大きな問題はないこと、個別訓練を週2回実施していることとする。	Participants don't have progressive disease, constitutional symptoms are stable, communication and a cognitive function not having the major problem.

		They conducted individual training twice a week.
除外基準 (Key exclusion criteria)	MMSE10点未満の方、コミュニケーション能力に問題のある方とする。中止基準は、研究参加者やその家族が中止を希望した場合、または担当医によって研究が継続できないと判断された場合とする。	MMSE<10<br>Participants have difficulty in communication.<br>We will stop studying that the participant could not carry on occupational therapy as judged by a primary doctor or participant and their family hope for a cancellation.
目標参加者数 (Target sample size)	60	

責任研究者 (Research contact person)		
項目(Item)	日本語(Japanese)	英語(English)
責任研究者名 (Name of lead principal investigator)	長山洋史	Hirofumi Nagayama
所属組織 (Organization)	神奈川県立保健福祉大学	Kanagawa University of Human Services
所属部署 (Division name)	作業療法学専攻	Department of occupational therapy
住所 (Address)	横須賀市平成町1-10-1	1-10-1 Heiseityou,Yokosuka
電話 (TEL)	0468282805	
Email (Email)	nagayama-ktr@kuhs.ac.jp	

試験問い合わせ窓口(Public contact)		
項目(Item)	日本語(Japanese)	英語(English)
担当者名 (Name of contact person)	長山洋史	Hirofumi Nagayama
組織名 (Organization)	神奈川県立保健福祉大学	Kanagawa University of Human Services
部署名	作業療法学専攻	Department of occupational therapy

(Division name)		
住所<br>(Address)	横須賀市平成町1-10-1	1-10-1 Heiseityou,Yokosuka
電話<br>(TEL)	0468282805	
試験のホームページURL<br>(Homepage URL)		
Email<br>(Email)	nagayama-ktr@kuhs.ac.jp	

実施責任組織 (Sponsor)		
項目(Item)	日本語(Japanese)	英語(English)
実施責任組織<br>(Name of primary sponsor)	神奈川県立保健福祉大学	Kanagawa University of Human Services

実施責任組織とは、「試験の計画、解析と結果公表、研究費調達を含めた実施のための運営管理に対して責任を持つ組織」です。英語名でスポンサーとありますが、通常イメージする資金提供者のことではございません。従いまして、「なし」という記載はありえません。

研究費提供組織(Funding Source)		
項目(Item)	日本語(Japanese)	英語(English)
研究費提供組織<br>(Source of funding)	日本作業療法士協会	Japanese Assosiation of Occupational Therapists
組織の区分<br>(Category of Org.)	その他/Other	
研究費拠出国<br>(Nation of funding)		

その他の関連組織 (Other related organizations)		
項目(Item)	日本語(Japanese)	英語(English)

共同実施組織 (Co-sponsor)		
その他の研究費提供組織 (Name of secondary fund er(s))		

他機関から発行された試験ID (Secondary study IDs)		
項目(Item)	日本語(Japanese)	英語(English)
他機関から発行された試験ＩＤ (Secondary study IDs)	いいえ/NO	
試験ID1 (Secondary study ID_1)		
ID発行機関1 (Org. issuing Secondary study ID_1)		
試験ID2 (Secondary study ID_2)		
ID発行機関2 (Org. issuing Secondary study ID_2)		
治験届 (IND to MHLW)		

試験実施施設 (Institutions)		
項目(Item)	日本語(Japanese)	英語(English)
試験実施施設名称 (Institutions)		

試験進捗状況 (Progress)		
項目(Item)	日本語(Japanese)	英語(English)

試験進捗状況 (Recruitment status)	試験終了/Completed
プロトコル確定日 (Date of protocol fixation)	2013/04/01
登録・組入れ開始（予定）日 (Anticipated trial start date)	2013/09/01
フォロー終了(予定)日 (Last follow-up date)	2014/02/28
入力終了(予定)日 (Date of closure to data entry)	2014/03/31
データ固定（予定）日 (Date trial data considered complete)	2014/06/01
解析終了(予定)日 (Date analysis concluded)	2014/08/01

関連情報 (Related information)		
項目(Item)	日本語(Japanese)	英語(English)
プロトコル掲載URL (URL releasing protocol)		
試験結果の公開状況 (Publication of results)	未公表/Unpublished	
結果掲載URL (URL releasing results)		
主な結果 (Results)		
その他関連情報		

(Other related information)		

管理情報		
項目(Item)	日本語(Japanese)	英語(English)
登録日 (Date of registration)	2014/01/28	
最終情報更新日 (Date of last update)	2014/09/10 15:12:22	

閲覧ページへのリンク	
日本語URL	https://upload.umin.ac.jp/cgi-open-bin/ctr/ctr.cgi?function=brows&action=brows&recptno=R000014739&type=summary&language=J
英語URL	https://upload.umin.ac.jp/cgi-open-bin/ctr/ctr.cgi?function=brows&action=brows&recptno=R000014739&type=summary&language=E

※ 本ページ収載の情報は、臨床試験に関する情報公開を目的として、UMINが開設しているUMIN臨床試験登録システムに提供された臨床試験情報です。

※ 特定の医薬品や治療法等については、医療関係者や一般の方に向けて広告することは目的としていません。

戻る

UMIN臨床試験登録システムのご使用に関するお問い合わせは、こちらのお問い合わせフォーム からお願いいたします。それ以外のお問い合わせは、 こちら よりお願い致します。

UMIN Infrastructure for Academic Activities
University hospital Medical Information Network
